# Long non‐coding RNA MALAT1 triggers ferroptosis via interaction with FUS to enhance ACSF2 mRNA stabilization in septic acute kidney injury

**DOI:** 10.1002/kjm2.12898

**Published:** 2024-10-10

**Authors:** Zhi‐Bing Duan, Ji‐Fu Zheng, Si‐Yue Huang, Li‐Li Hu

**Affiliations:** ^1^ Department of Nephrology The Second Affiliated Hospital, Jiangxi Medical College, Nanchang University Nanchang Jiangxi Province China; ^2^ Department of Hematology The Second Affiliated Hospital, Jiangxi Medical College, Nanchang University Nanchang Jiangxi Province China

**Keywords:** ACSF2, ferroptosis, FUS, lncRNA MALAT1, septic acute kidney injury

## Abstract

Septic acute kidney injury (AKI) is a fatal disease in the intensive care unit, with ferroptosis playing a crucial role in its pathogenesis. Long non‐coding RNA (LncRNA) metastasis‐associated lung adenocarcinoma transcript 1 (MALAT1) has been implicated in septic‐induced AKI inflammation and apoptosis. However, its regulatory role in ferroptosis and underlying mechanisms remain unclear. In vivo and in vitro models of septic AKI were established using cecal ligation and puncture (CLP) and lipopolysaccharide (LPS) challenge, respectively. Serum levels of creatinine (Cr), blood urea nitrogen (BUN), kidney injury molecule‐1 (Kim‐1), neutrophil gelatinase‐associated lipocalin (NGAL), and inflammatory cytokine in kidney tissues were determined by ELISA kits. Histopathological alterations and apoptosis were evaluated by HE staining and TUNEL. Ferroptosis was accessed by measuring MDA, GSH, Fe^2+^, total and lipid ROS levels, and mitochondrial ultrastructure changes. Target molecular levels were determined using RT‐qPCR, Western blotting, and immunofluorescence. Interactions among MALAT1, acyl‐CoA synthetase family member 2 (ACSF2) and FUS RNA binding protein (FUS) were validated by RIP and RNA‐pull down. MALAT1 level was significantly elevated in both in vivo and in vitro septic AKI models, of which knockdown impeded ferroptosis to alleviate septic AKI. Mechanistically, high MALAT1 expression increased ACSF2 mRNA stability via interaction with FUS. Rescue experiments showed that ACSF2 overexpression partially reversed the ferroptosis inhibition mediated by MALAT1 silencing. MALAT1 induces ferroptosis and exacerbates septic AKI by stabilizing ACSF2 mRNA with the assistance of FUS. These findings provide theoretical evidence for MALAT1 as a potential therapeutic target for septic AKI.

**Abbreviations**
ACSF2: synthetase family member 2
AKI: acute kidney injury
ATCC: American Type Cell Culture
BUN: blood urea nitrogen
CFU: colony forming unit
CLP: cecal ligation and puncture
Cr: creatinine
ELISA: enzyme‐linked immunosorbent assay
Fe^2+^
: ferrous ion
FUS: acyl‐CoA FUS RNA binding protein
GSH: glutathione
HE: hematoxylin and eosin
KIM‐1: kidney injury molecule 1
lncRNAs: long non‐coding RNAs
LV: lentiviruses
LV‐shMALAT1: lentiviruses carrying shMALAT1
MALAT1: metastasis associated lung adenocarcinoma transcript 1
MDA: malondialdehyde
NGAL: neutrophil gelatinase‐associated lipocalin
RBPs: RNA binding proteins
RT‐qPCR: real‐time quantitative PCR
RIP: RNA immunoprecipitation
SD: standard deviation
TEM: transmission electron microscopy
TUNEL: terminal‐deoxynucleoitidyl transferase mediated nick end labeling

## 1 INTRODUCTION

Sepsis is a severe inflammatory response syndrome caused by an uncontrolled host response to infection, leading to multiple organ dysfunction.^1^ It is a leading cause of death among intensive care unit patients. Acute kidney injury (AKI) is one of the major complications of sepsis, occurring in approximately 60% of septic patients.^2^ The prevalence and mortality rates of septic AKI patients increase progressively with the severity of sepsis.^3^ The pathogenesis of septic AKI is complex and not fully understood, and there is still a lack of effective therapies. Therefore, it is essential to uncover the potential pathological mechanisms and develop novel therapeutic targets for septic AKI.

Long non‐coding RNAs (lncRNAs) are RNA molecules longer than 200 nucleotides that do not encode proteins. Mounting evidence suggests that the dysregulated lncRNAs participate in the development of multiple diseases, including septic AKI.^4^ For instance, lncRNA NONRATG019935.2 is downregulated during sepsis‐induced AKI, and its overexpression alleviates septic AKI through inhibiting renal tubular epithelial cell apoptosis.^5^ LncRNA metastasis‐associated lung adenocarcinoma transcript 1 (MALAT1) is highly expressed in the rat model of septic AKI, and inhibiting MALAT1 expression contributes to the therapeutic effects of resveratrol or paclitaxel in septic AKI.^6^, ^7^ However, the detailed regulatory mechanisms of MALAT1 in the pathological development of septic AKI remain unclear and warrant further investigation.

Ferroptosis is a newly identified form of programmed cell death closely related to iron metabolism, characterized by GSH depletion and excessive lipid ROS production.^8^ Excessive ROS production may cause intracellular oxidative stress injury, leading to damages of proteins, DNAs, RNAs, lipids, and ultimately inducing ferroptosis.^9^ During ferroptosis, mitochondria shrink, accompanied by increased mitochondrial membrane density and the disappearance of mitochondrial cristae.^10^ Recent studies have shown that ferroptosis is involved in the pathogenesis of septic AKI. For example, Liang et al. reported that mitochondrial ROS induced ferroptosis in renal cells upon LPS stimulation.^11^ Suppression of ferroptosis has been shown to protect against septic AKI.^12^, ^13^ However, the role of ferroptosis in MALAT1‐mediated regulation of septic AKI has not been clarified.

In this study, we aimed to explore the modulation of MALAT1 in ferroptosis during septic AKI and its underlying mechanism. Our findings showed that MALAT1 is aberrantly upregulated in sepsis‐induced AKI models. Downregulation of MALAT1 restrained sepsis‐induced ferroptosis in renal cells by recruiting the RNA binding protein FUS to reduce the stability of acyl‐CoA synthetase family member 2 (ACSF2) mRNA. This study sheds light on novel mechanism through which MALAT1 contributes to septic AKI progression, suggesting MALAT1 as a potential therapeutic target for septic AKI.

## 2 MATERIALS AND METHODS

### 2.1 Ethics statement

The animal experiments followed the Guide for the Care and Use of Laboratory Animals (NIH Publication, 8th Edition, 2011). All experimental procedures were approved by the Ethic Committee of The Second Affiliated Hospital of Nanchang University.

### 2.2 Animal model

Male C57BL/6 mice (8–10 weeks old) were purchased from SLAC Laboratory Animal Co., Ltd. (Shanghai, China). Four experimental groups were established (*n* = 8 per group): sham, cecal ligation and puncture (CLP), CLP + lentiviruses carrying shNC (LV‐shNC), and CLP + LV‐shMALAT1. LV‐shMALAT1 or LV‐shNC (2 × 10^7^ TU, GeneChem, Shanghai, China) were injected into the mice via tail veins. Five days post‐injection, septic AKI was induced by CLP as previously described.^14^ Briefly, a midline incision was made in the anesthetized mice on the abdomen to expose the cecum, which was ligated with a 4‐0 suture and penetrated twice with a puncture needle, followed by abdominal wound closure. Sham mice underwent the same surgical procedures without CLP.

### 2.3 Bacterial load analysis

Blood samples were collected 12 h post‐surgery, diluted in sterile PBS at a1:3 ratio. Subsequently, 100 μL of diluted blood was plated on 5% blood agar plates. After incubation for 24 h at 37°C, the colony forming unit (CFU) was counted.

### 2.4 Measurement of serum creatinine, blood urea nitrogen

Serum levels of creatinine (Cr) and blood urea nitrogen (BUN) were measured using commercial Cr and BUN assay kits purchased from Jiancheng Bioengineering Institute (Nanjing, China) according to manufacturer's protocols.

### 2.5 Histological examination

Kidney tissues of mice were fixed in 4% paraformaldehyde, embedded in paraffins, and sliced into 5 μm sections. Pathological alterations were observed using hematoxylin and eosin (HE) staining with an HE staining kit (Boster, Wuhan, China). Images were captured under a light microscope (Olympus, Japan). Renal injury scores were assessed by a pathologist blinded to the experiment, as previously described.^15^

### 2.6 Terminal‐deoxynucleoitidyl transferase mediated nick end labeling

Terminal‐deoxynucleoitidyl transferase mediated nick end labeling (TUNEL) assay was conducted to assess apoptosis in the kidney sections using the TUNEL Assay Kit‐HRP kit (Abcam, UK) according to the manufacturer's instructions. The percentage of apoptosis was quantified in 10 random fields using Image J software.

### 2.7 Enzyme‐linked immunosorbent assay

Levels of TNF‐α, IL‐6, and IL‐1β in kidney tissues, and serum neutrophil gelatinase‐associated lipocalin (NGAL) and kidney injury molecule 1 (KIM‐1) levels were measured using commercial mouse TNF‐alpha Quantikine ELISA Kit (R&D Systems, USA), mouse IL‐6 Quantikine ELISA Kit (R&D Systems), mouse IL‐1 beta/IL‐1F2 Quantikine ELISA Kit (R&D Systems), mouse Lipocalin‐2/NGAL Quantikine ELISA Kit (R&D Systems), and mouse TIM‐1/KIM‐1/HAVCR Quantikine ELISA Kit (R&D Systems), respectively.

### 2.8 Transmission electron microscopy

Renal tissues were fixed with 2.5% glutaraldehyde at 4°C overnight, followed by paraffin embedding and slicing into 60–80 nm sections. Mitochondria morphology was examined under a TEM (HITACHI, Japan).

### 2.9 Cell culture and transfection

Human HK‐2 cells were purchased from American Type Cell Culture (ATCC, USA). HK‐2 cells were maintained in DMFM/F‐12 (Gibco, USA) containing 10% fetal bovine serum (Gibco) at 37°C with 5% CO_2_. To mimic septic AKI in vitro, HK‐2 cells were exposed to LPS (2.5, 5, 10 μg/mL, Solarbio, Beijing, China) for 24 h. For cell transfection, HK‐2 cells at 70% confluence were transfected with ACSF2 overexpression plasmid or vector (GeneChem) using Lipofectamine 2000 (Thermo Fisher, USA).

### 2.10 Lentivirus infection

HK‐2 cells were infected with lentiviruses carrying shMALAT1 (LV‐shMALAT1), or LV‐shNC (Genechem) at a multiplicity of infection (MOI) of 50 in the presence of 5 μg/mL polybrene.

### 2.11 CCK‐8 assay

HK‐2 cells were seeded into 96‐well plates (4 × 10^3^ cells/well). After overnight culture, 10 μL of CCK‐8 solution (Beyotime, Haimen, China) was added and incubated for 2 h. Corresponding absorbance was measured at 450 nm on a microplate reader (Thermo Fisher).

### 2.12 Detection of ROS level

Intracellular ROS level was measured using DCFH‐DA staining. Briefly, HK‐2 cells were stained with 10 μM DCFH‐DA solution (Solarbio) for 30 min. The stained cells were washed with PBS to remove the residual DCFH‐DA solution and observed under a fluorescence microscope (Olympus).

### 2.13 Measurement of malondialdehyde, glutathione, and ferrous ion

The levels of glutathione (GSH), malondialdehyde (MDA), and ferrous ion (Fe^2+^) were assessed using the Reduced GSH Content Assay Kit (Solarbio), MDA Content Assay Kit (Solarbio), Ferrous Ion Content Assay Kit (Solarbio) following the manufacturer's instructions, respectively.

### 2.14 Detection of lipid ROS

Lipid ROS levels were measured using the C11‐BODIPY 581/591 probe (Thermo Fisher). Briefly, HK‐2 cells were stained with 2 μM C11‐BODIPY 581/591 in serum‐free medium at 37°C for 30 min. Subsequently, the stained HK‐2 cells were analyzed by flow cytometry.

### 2.15 Cytoplasmic and nuclear separation

The PARISTM Kit (Thermo Fisher) was utilized to isolate cytoplasmic and nuclear fractions. In brief, HK‐2 cells were treated with the Cell Disruption Buffer for 10 min. Subsequently, the cytoplasmic and nuclear fractions were separated.

### 2.16 Real‐time quantitative PCR

Total RNA from kidney tissues, cells, cytoplasmic, and nuclear fractions was isolated using the TRIzol reagent (Thermo Fisher). cDNA was synthesized using the FastKing cDNA synthesis kit (TIANGEN, Beijing, China). qPCR was conducted using the Talent qPCR PreMix (SYBR Green) (TIANGEN). Relative mRNA levels were normalized to GAPDH and quantified using the 2^−ΔΔCt^ method. Primer sequences are listed in Table 1.

**TABLE 1:** Oligonucleotide primer sets for RT‐qPCR.

Name	Sequence (5′ → 3′)	Length
Human MALAT1 F	GTAACGATGGTGTCGAGGTC	20
Human MALAT1 R	CAGCATTACAGTTCTTGAACATG	23
Mouse MALAT1 F	GGCAGAATGCCTTTGAAGAG	20
Mouse MALAT1 R	GGTCAGCTGCCAATGCTAGT	20
Human ACSF2 F	ATGAGAAGACACCAGAGCAGTT	22
Human ACSF2 R	GCACCGTACATCAGACACATC	21
Human FUS F	ATGGCCTCAAACGATTATACCCA	23
Human FUS R	GTAACTCTGCTGTCCGTAGGG	21
Human GAPDH F	GGTGTGAACCATGAGAAGTATGA	23
Human GAPDH R	GAGTCCTTCCACGATACCAAAG	22
Mouse GAPDH F	CACTGAGCAAGAGAGGCCCTAT	22
Mouse GAPDH R	GCAGCGAACTTTATTGATGGTATT	24

### 2.17 Western blotting

Protein from the cytoplasm and mitochondria was extracted using the Cell Mitochondria Isolation Ki (Beyotime). Protein concentration was quantified using the BCA Protein Assay Kit (Beyotime). Protein samples (15 μg) were subjected to sodium dodecyl sulfate polyacrylamide gel (SDS‐PAGE) electrophoresis and transferred to the polyvinylidene difluoride (PVDF) membranes. The membranes were blocked with 5% skim milk for 1 h and probed with primary antibodies against ACSF2 (1:500, 16140‐1‐AP, Proteintech, Wuhan, China), FUS (1:1000, 11570‐1‐AP, Proteintech), COX IV (1:1000, 11242‐1‐AP, Proteintech), GAPDH (1:5000, 10494‐1‐AP, Proteintech) at 4°C overnight. Subsequently, the membranes were incubated with HRP Goat Anti‐Rabbit IgG (AS014, 1:2000, ABclonal), and the protein bands were visualized using ECL Western Blotting Substrate (Solarbio).

### 2.18 Immunofluorescence staining

HK‐2 cells were stained with 500 nM MitoTracker deep red FM solution (Thermo Fisher) for 20 min, fixed with 4% paraformaldehyde, and permeabilized with 0.1% Triton X‐100 for 10 min. After blocking with 5% BSA for 1 h, the cells were incubated with anti‐ACSF2 (1:20, 16140‐1‐AP, Proteintech) primary antibodies at 4°C overnight, followed by FITC‐conjugated secondary antibody (1:1000, ab6717, Abcam). The nuclei were counterstained with DAPI. Finally, the localization of ACSF2 in mitochondria was observed under a fluorescence microscope.

### 2.19 RNA immunoprecipitation

RNA immunoprecipitation (RIP) assay was performed to verify the interaction between FUS and MALAT1/ACSF2. Lysates from HK‐2 cells were isolated with RIP lysis buffer and immunoprecipitated with protein A/G beads conjugated with anti‐FUS (11570‐1‐AP, Proteintech) or anti‐IgG (30000‐0‐AP, Proteintech) at 4°C. Immunocomplexes were eluted and treated with proteinase K. The enrichment of MALAT1 and ACSF2 mRNA in purified immunocomplexes was evaluated by RT‐qPCR.

### 2.20 RNA pull‐down assay

Protein extracts from HK‐2 cells were incubated with biotinylated MALAT1 and ACSF2 probes (Biomics Biotechnology, Beijing, China) at 37°C overnight. Immunocomplexes were extracted with the streptavidin magnetic beads for 4 h. Finally, the pull‐down complexes were further assessed by western blotting.

### 2.21 RNA stability assay

The stability of ACSF2 mRNA in HK‐2 cells was evaluated. After treatment with 5 μg/mL actinomycin D (Sigma–Aldrich, USA) for 2, 4, 6, 8, and 10 h, total RNA was extracted and the level of ACSF2 was detected by RT‐qPCR.

### 2.22 Statistical analysis

All data from at least three independent experiments are expressed as mean ± standard deviation (SD). Comparisons were performed using Student's *t*‐test or one‐way ANOVA followed by Bonferroni post hoc test where appropriate, using SPSS 19.0 software. *p* < 0.05 was considered statistically significant.

## 3 RESULTS

### 3.1 MALAT1 was overexpressed in CLP‐induced AKI in mice

**FIGURE 1:**
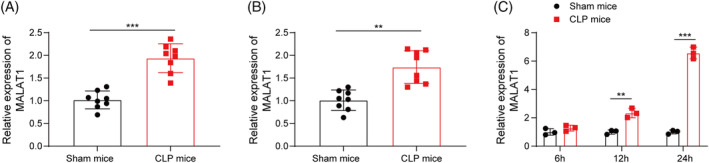
Up‐regulation of MALAT1 during septic AKI in mice. (A) RT‐qPCR analysis of MALAT1 expression in plasma samples from sham and CLP groups. (B) RT‐qPCR analysis of MALAT1 level in the kidneys of sham and CLP mice. (C) MALAT1 level at 6, 12, and 24 h in the kidney tissues after CLP was detected by RT‐qPCR. **p < 0.01, ***p < 0.001. *n* = 8.

First, the differential expression of MALAT1 between sham and CLP groups was evaluated. RT‐qPCR showed that MALAT1 expression was significantly higher in the serum and kidney tissues of septic mice compared with sham mice (Figure 1A,B). Furthermore, MALAT1 expression increased in a time‐dependent manner in the kidneys after CLP (Figure 1C). Therefore, MALAT1 was upregulated of in the kidney tissues of septic mice.

### 3.2 MALAT1 knockdown attenuated septic AKI in mice

**FIGURE 2:**
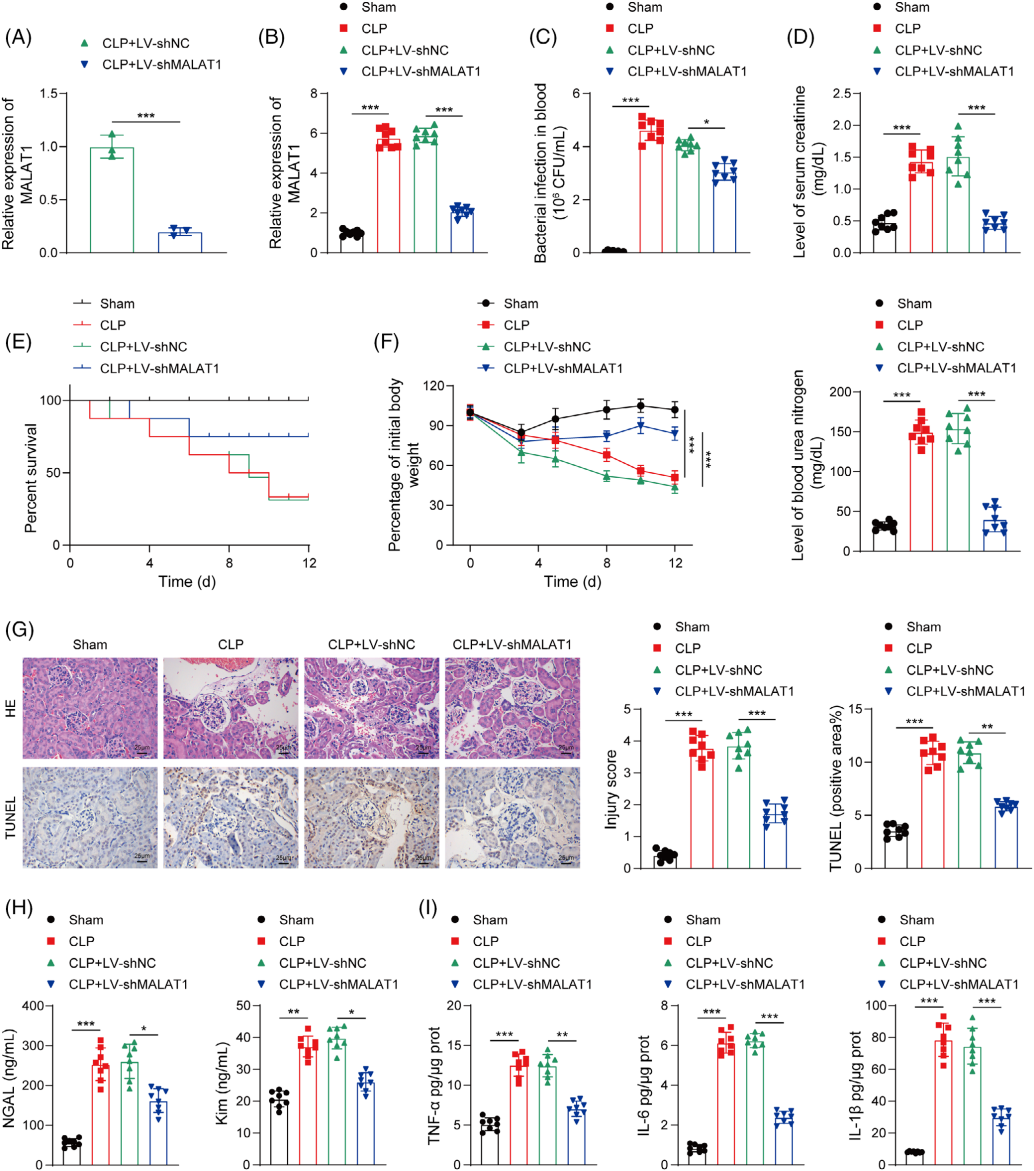
MALAT1 knockdown exhibited protective effects against inflammatory damage and renal injury in CLP‐induced AKI in mice. (A) The silencing efficiency of LV‐shMALAT1 was verified by RT‐qPCR. (B) MALAT1 expression in the kidneys of mice from different groups was assessed by RT‐qPCR. (C) Blood bacterial content of mice was detected. (D) The serum levels of Cr and BUN were measured. (E) Survival curve of mice from the indicated groups. (F) Body weight of mice was monitored. (G) The pathological changes and apoptosis in the kidney tissues were determined by HE staining and TUNEL, respectively. (H) The serum levels of NGAL and Kim‐1 were measured by ELISA. (I) ELISA assay for detection of TNF‐α, IL‐6, and IL‐1β levels in the kidney tissues. *p < 0.05, **p < 0.01, ***p < 0.001. *n* = 8.

To explore the biological function of MALAT1 in septic AKI, the CLP‐challenged mice were injected with LV‐shMALAT1. The silencing efficiency of MALAT1 was verified by RT‐qPCR (Figure 2A). In addition, the increased MALAT1 expression in the kidney tissues of septic mice was reversed by LV‐shMALAT1 injection (Figure 2B). The blood bacterial content of septic mice was evidently enhanced, which was reduced by MALAT1 knockdown (Figure 2C). Besides, CLP led to obvious renal injury, evidenced by increased serum Cr and BUN levels; these changes were mitigated after MALAT1 downregulation (Figure 2D). Notably, the survival rate of septic mice dramatically declined compared to that of sham mice; however, MALAT1 deficiency significantly enhanced the survival rate of septic mice (Figure 2E). Additionally, MALAT1 silencing remarkably improved the weight loss of septic mice (Figure 2F). Histological examination demonstrated that MALAT1 depletion attenuated tubulointerstitial injury, inflammatory cell infiltration, and apoptosis in the kidneys of septic mice, as well as reduced renal tubular damage score (Figure 2G). Moreover, the elevated NGAL and KIM‐1 levels in serum, TNF‐α, IL‐6, and IL‐1β levels in the kidneys in sepsis group were partly reversed by LV‐shMALAT1 (Figure 2H,I). These observations suggested that MALAT1 silencing effectively relieved sepsis‐induced inflammation and kidney damage in vivo.

### 3.3 MALAT1 depletion restrained ferroptosis induction in CLP‐induced mouse model of AKI

**FIGURE 3:**
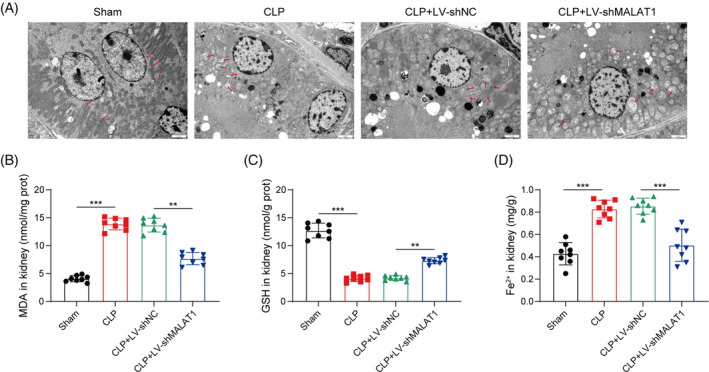
MALAT1 down‐regulation repressed CLP‐induced ferroptosis in the kidneys of mice. (A) TEM observed the ultrastructural changes of mitochondria. (B–D) The levels of MDA, GSH, and Fe^2+^ were measured by commercial kits. *p < 0.05, **p < 0.01, ***p < 0.001. *n* = 8.

Since ferroptosis is implicated in the pathogenesis of septic AKI, we further investigated the regulation of MALAT1 in ferroptosis. TEM images revealed that sepsis‐induced mitochondrial shrinking and mitochondrial crest reduction in the kidney tissues were abolished by MALAT1 deficiency (Figure 3A). Additionally, the elevated MDA and Fe^2+^ levels and reduced GSH level in septic mice, were reversed after MALAT1 silencing (Figure 3B–D). These findings indicated that MALAT1 knockdown inhibited ferroptosis during septic AKI development.

### 3.4 Silencing of MALAT1 mitigated LPS‐induced ROS accumulation and ferroptosis in HK‐2 cells

**FIGURE 4:**
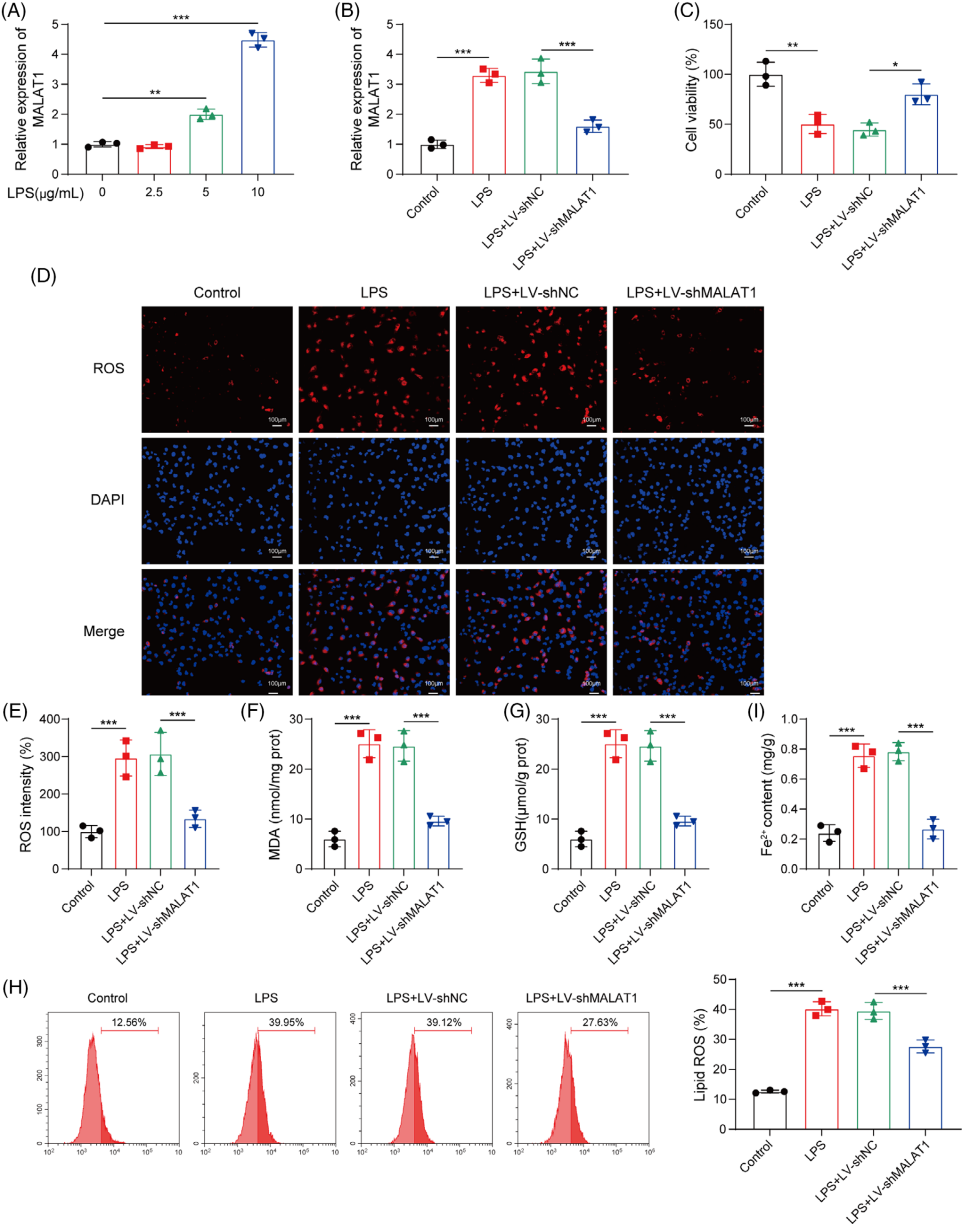
MALAT1 depletion mitigated ferroptosis in LPS‐stimulated HK‐2 cells. (A) RT‐qPCR analysis of MALAT1 level after exposure to different concentrations of LPS in HK‐2 cells. LPS‐treated HK‐2 cells were infected with LV‐shNC or LV‐shMALAT1. (B) MALAT1 expression in HK‐2 cells with various treatments was evaluated by RT‐qPCR. (C) HK‐2 cell viability was assessed by CCK‐8 assay. (D and E) Intracellular ROS level in HK‐2 cells was determined by staining with DCFH‐DA probe. (F and G) MDA and GSH levels in HK‐2 cells were measured. (H) Lipid ROS level was analyzed using C11‐BODIPY 581/591 probe by flow cytometry. (I) Fe^2+^ level in HK‐2 cells was detected. *p < 0.05, **p < 0.01, ***p < 0.001. *n* = 3.

We further aimed to confirm the modulation of MALAT1 in ferroptosis in LPS‐challenged HK‐2 cells in vitro. We found a dose‐dependent increase in MALAT1 expression in HK‐2 cells after LPS exposure (Figure 4A). In addition, the LPS‐induced upregulation of MALAT1 was abolished by LV‐shMALAT1 infection (Figure 4B). The CCK‐8 assay showed that LPS exposure strikingly decreased HK‐2 cell viability; however, the declined cell viability was recovered in MALAT1‐depleted group (Figure 4C). Additionally, the enhanced release of ROS from LPS‐stimulated HK‐2 cells was weakened by MALAT1 inhibition (Figure 4D,E). Moreover, the MDA level was enhanced, while GSH level was reduced by LPS challenge; MALAT1 deficiency reversed these alterations (Figure 4F,G). Flow cytometry analysis showed that lipid ROS levels were enhanced by LPS stimulation, which were reversed in MALAT1‐silenced cells (Figure 4H). Additionally, MALAT1 knockdown abolished LPS‐mediated elevation in Fe^2+^ level (Figure 4I). The above results revealed that MALAT1 knockdown restrained LPS‐triggered ferroptosis in vitro.

### 3.5 MALAT1 facilitated mitochondrial localization and expression of ACSF2

**FIGURE 5:**
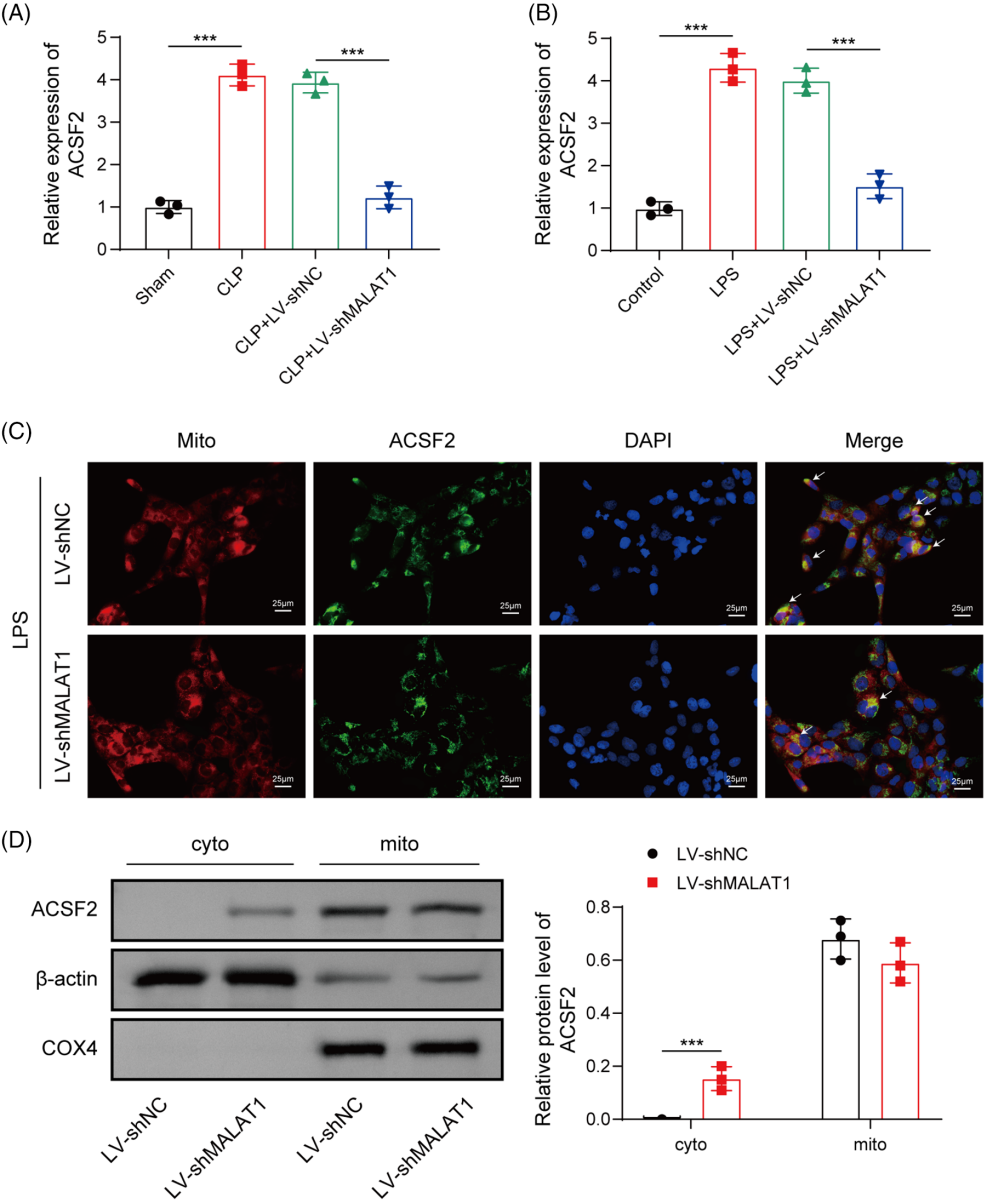
MALAT1 affected subcellular expression of ACSF2. (A) ACSF2 expression in the kidneys of mice from different groups was measured by RT‐qPCR. (B) ACSF2 expression in HK‐2 cells with various treatments was detected by RT‐qPCR. (C) Double‐immunofluorescence staining of ACSF2 and MitoTracker Deep Red FM in HK‐2 cells with or without MALAT1 knockdown. White arrows indicate double dyed areas. (D) Western blotting analyzed the expression of ACSF2 protein in cytoplasm and mitochondria of HK‐2 cells. COX4 and β‐Actin served as loading controls for the mitochondrial and cytoplasmic fractions, respectively. *p < 0.05, ***p < 0.001. For A, *n* = 8; for B–D, *n* = 3.

Mitochondria, key organelles of oxidative metabolism, have been recognized to participate in ferroptosis through regulation of Fe^2+^ and energy metabolism homeostasis.^16^ ACSF2, mainly expressed in mitochondria, has been reported to facilitate ischemia–reperfusion‐induced AKI via repressing mitophagy.^17^ Therefore, we further explored whether MALAT1 affected ferroptosis during sepsis‐induced AKI through regulation of ACSF2. ACSF2 expression was increased in the kidney tissues of septic mice and LPS‐stimulated HK‐2 cells, and MALAT1 knockdown abolished this elevation (Figure 5A,B). Double‐immunofluorescent staining showed that ACSF2 was mainly localized in the mitochondria of LPS‐exposed HK‐2 cells, which was attenuated after MALAT1 knockdown (Figure 5C). Furthermore, Western blotting demonstrated that MALAT1 depletion promoted the translocation of ACSF2 protein from the mitochondria to cytoplasm of HK‐2 cells (Figure 5D). Thus, MALAT1 contributed to the mitochondrial expression of ACSF2 in LPS‐stimulated HK‐2 cells.

### 3.6 Enforced expression of ACSF2 abolished MALAT1 silencing‐mediated biological functions in HK‐2 cells

**FIGURE 6:**
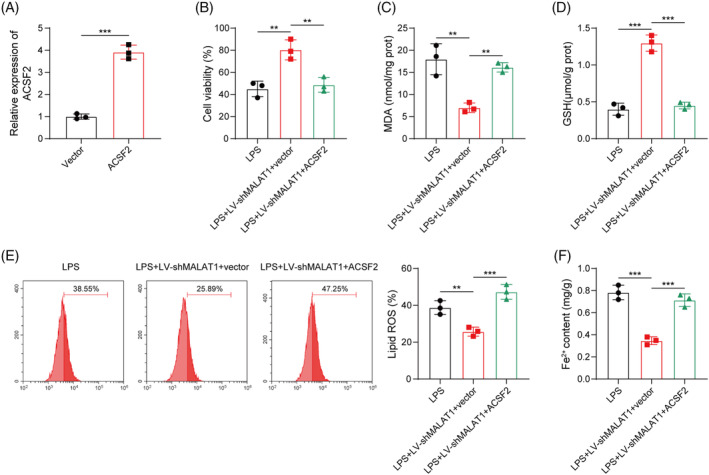
Overexpression of ACSF2 counteracted MALAT1 silencing‐mediated biological functions in HK‐2 cells. (A) RT‐qPCR analysis of ACSF2 mRNA level after transfection with OE‐ACSF2. HK‐2 cells were transduced with LV‐shMALAT1 combined with or without OE‐ACSF2 upon LPS exposure. (B) CCK‐8 assay detected cell viability. (C and D) MDA and GSH contents were detected by commercial kits. (E) Lipid ROS level was assessed using C11‐BODIPY 581/591 probe by flow cytometry. (F) Fe^2+^ level in HK‐2 cells was detected. **p < 0.01, ***p < 0.001. *n* = 3.

To verify the involvement of MALAT1/ACSF2 axis in the regulation of ferroptosis, an ACSF2 overexpression plasmid was co‐transfected into shMALAT1‐transfected HK‐2 cells. The overexpression efficiency of ACSF2 in HK‐2 cells was confirmed by RT‐qPCR (Figure 6A). The CCK‐8 assay indicated that the elevated cell viability of MALAT1‐silenced cells was abolished by ACSF2 overexpression (Figure 6B). Furthermore, ACSF2 overexpression partly reversed shMALAT1‐induced decreased MDA level and increased GSH level in LPS‐stimulated HK‐2 cells (Figure 6C,D). Additionally, the declined lipid ROS production and enhanced Fe^2+^ level mediated by MALAT1 depletion upon LPS exposure was antagonized by overexpression of ACSF2 (Figure 6E,F). As shown in Figure S1A, ACSF2 depletion strikingly enhanced the viability of LPS‐stimulated HK‐2 cells. Moreover, ACSF2 knockdown reduced MDA, lipid ROS, and Fe^2+^ levels, but increased GSH level in LPS‐stimulated HK‐2 cells (Figure S1B–F). Collectively, MALAT1 knockdown repressed LPS‐triggered ferroptosis of HK‐2 cells by inhibiting ACSF2 expression.

### 3.7 MALAT1 interacted with FUS to enhance ACSF2 mRNA stability

**FIGURE 7:**
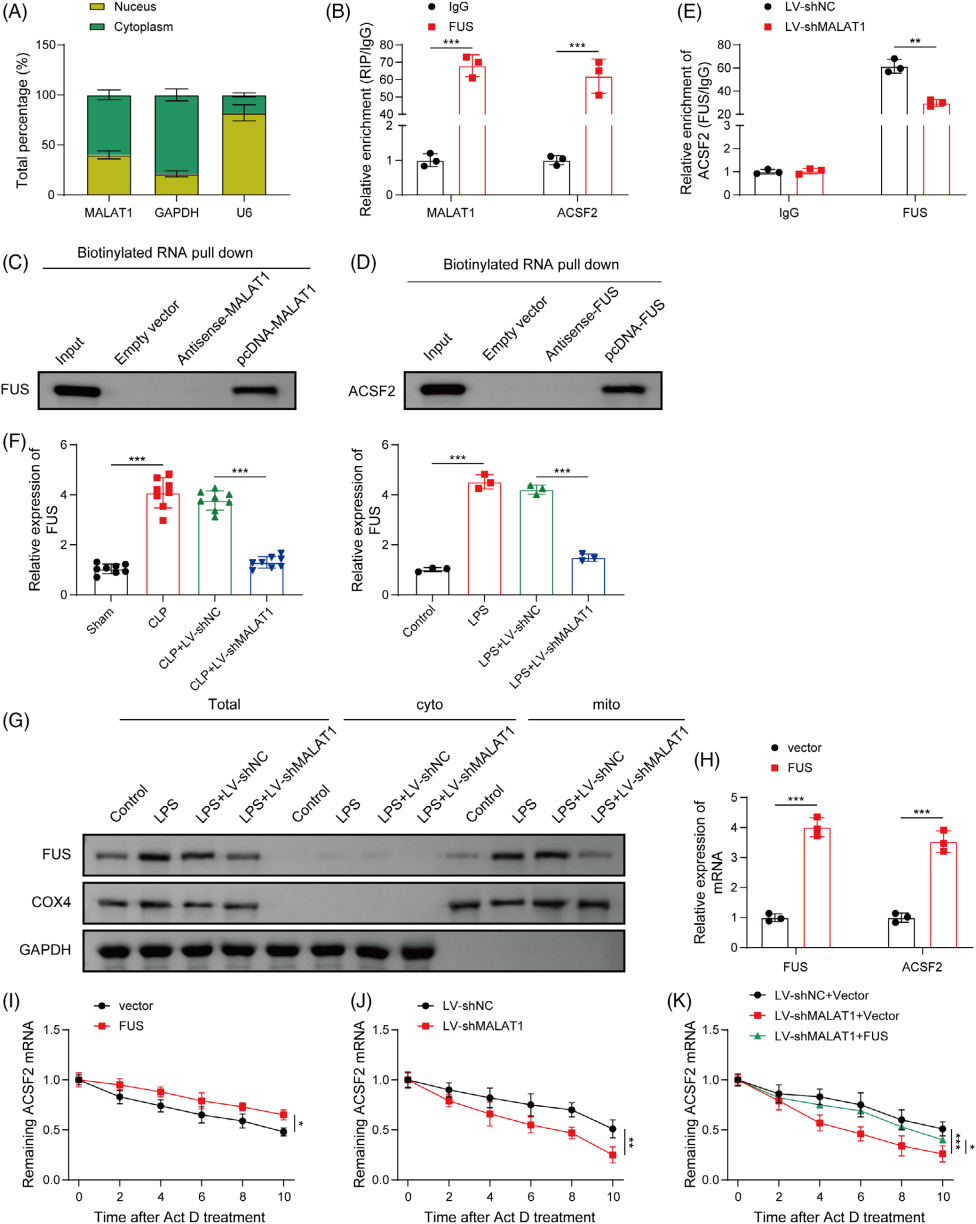
MALAT1 enhanced ACSF2 mRNA stability via recruitment of FUS. (A) The subcellular distribution of MALAT1 was evaluated by RT‐qPCR. RIP (B) and RNA pull‐down assay (C and D) validated the direct binding of MALAT1/ACSF2 to FUS. (E) RIP assay verified the interaction between FUS protein and ACSF2 mRNA in HK‐2 cells infected with LV‐shNC or LV‐shMALAT1. (F) FUS mRNA level in kidneys or HK‐2 cells from different groups was measured by RT‐qPCR. (G) Western blotting analyzed FUS protein level in cytoplasm and mitochondria of LPS‐exposed HK‐2 cells infected with LV‐shNC or LV‐shMALAT1. (H) HK‐2 cells were transfected with vector or FUS overexpression plasmid, and FUS and ACSF2 mRNA levels were assessed by RT‐qPCR. (I) ACSF2 mRNA stability of HK‐2 cells transfected with vector or FUS overexpression plasmid was determined by RT‐qPCR. (J) ACSF2 mRNA stability of HK‐2 cells infected with LV‐shNC or LV‐shMALAT1 was assessed by RT‐qPCR. (K) HK‐2 cells were infected with LV‐shNC or LV‐shMALAT1 together with or without transfection with vector or FUS overexpression plasmid, and ACSF2 mRNA stability was detected by RT‐qPCR. **p < 0.01, ***p < 0.001. *n* = 3.

Next, we sought to clarify the potential mechanism through which MALAT1 facilitated ACSF2 expression. The expression of MALAT1 in cytoplasmic and nuclear fractions was detected, revealing that MALAT1 was mainly expressed in the cytoplasm of HK‐2 cells (Figure 7A). Interestingly, bioinformatics analysis predicted that both MALAT1 and ACSF2 could bind to RNA‐binding protein FUS. RIP and RNA pull‐down assays validated that FUS protein could directly bind to MALAT1 and ACSF2 (Figure 7B–D). The direct binding of FUS to ACSF2 mRNA was weakened by MALAT1 knockdown (Figure 7E). RT‐qPCR analysis showed that FUS mRNA levels were evidently increased in the in vivo and in vitro models of septic AKI, whereas the elevated FUS expression was abolished by LV‐shMALAT1 injection (Figure 7F). However, FUS mRNA stability was not changed after MALTA1 silencing (Figure S2A). Western blotting results showed that FUS protein was mainly expressed in the mitochondria, but rarely in the cytoplasm of HK‐2 cells. This result was intensified after LPS stimulation, which was abrogated after MALAT1 knockdown (Figure 7G). FUS overexpression obviously enhanced ACSF2 mRNA level in HK‐2 cells (Figure 7H). Accordingly, ACSF2 mRNA stability in response to actinomycin D was strikingly raised by FUS overexpression (Figure 7I). Moreover, silencing of MALAT1 or FUS remarkably reduced ACSF2 mRNA stability (Figure 7J). MALAT1 overexpression remarkably enhanced ACSF2 mRNA expression and stability, which was counteracted by FUS knockdown (Figure S2B,C). shMALAT1‐mediated reduction in ACSF2 mRNA stability could be partly recovered by FUS overexpression (Figure 7K). To sum up, MALAT1 contributed to ACSF2 mRNA stability via interaction with FUS.

## 4 DISCUSSION

Sepsis is a life‐threatening disease often accompanied by multiple organ injuries, primarily caused by uncontrollable response to infection.^18^ As a major complication of sepsis, AKI may lead to high mortality and poor long‐term outcomes for sepsis patients.^19^ Despite its clinical significance, the complex pathogenesis of septic AKI remains poorly understood, and specific, effective therapies are lacking. In this work, we investigated the function and regulatory mechanism of lncRNA MALAT1 in septic AKI, both in vivo and in vitro. Genetic knockdown of MALAT1 protected mouse kidney and HK‐2 cells against sepsis via repressing ferroptosis. Mechanistically, MALAT1 enhanced ACSF2 mRNA stability through recruitment of the RNA‐binding protein FUS. Overexpression of ACSF2 counteracted the protective effects of MALAT1 silencing against LPS‐induced oxidative injury and ferroptosis in HK‐2 cell. Therefore, the MALAT1 inhibition could be a promising therapeutic strategy for treating septic AKI.

Ferroptosis is a novel type of cell death characterized by iron‐dependent lipid peroxidation, involves distinct morphological changes, such as mitochondrial shrinking coupled with increased membrane density.^20^ Oxidative damage and lipid peroxidation are critical contributors to ferroptosis.^21^ Recent studies have suggested that ferroptosis plays a key role in various diseases, including septic AKI.^22^ The lncRNA MALAT1 has been demonstrated to promote ROS release and oxidative stress injury in multiple disorders.^23^, ^24^ A previous study documented that MALAT1 promotes the inflammatory response in LPS‐challenged HK‐2 cells.^6^ Similarly, Ding et al. found that MALAT1 facilitated LPS‐induced AKI via sponging miR‐146a, thereby activating the NF‐κB pathway.^23^ However, whether MALAT1 modulates ferroptosis in septic AKI remained unclear. In this study, we found that MALAT1 is upregulated in sepsis‐induced AKI models. Moreover, MALAT1 depletion restrained sepsis‐triggered ferroptosis in renal cells, as evidenced by decreasing Fe^2+^, MDA, and lipid ROS levels, along with increased cell viability and GSH levels. These findings provide the first evidence that high expression of MALAT1 induces ferroptosis during the development of septic AKI.

Ferroptosis is close association with mitochondria function, as mitochondrial dysfunction has been shown to trigger ferroptosis through the abnormal accumulation of Fe^2+^.^24^ Fe^2+^ imbalance can lead to mitochondrial dysfunction via reducing the activities of electron transport chain enzymes, decreasing mitochondrial membrane potential, and enhancing ROS release.^25^ ACSF2 is a mitochondria protein that plays a critical role in fatty acid metabolism within mitochondria.^26^ Meanwhile, it has been identified as a ferroptosis‐related gene.^27^ In our study, we found that MALAT1 knockdown caused the translocation of ACSF2 from mitochondria to cytoplasm. Moreover, overexpression of ACSF2 counteracted the inhibitory effects of MALAT1 silencing on ferroptosis in LPS‐challenged HK‐2 cells. We further explored the potential mechanism by which MALAT1 increases ACSF2 expression. It is known that lncRNAs can regulate their target gene expression through direct interactions with RNA‐binding proteins.^28^ RNA‐binding proteins are capable of post‐transcriptionally regulating mRNA stability.^29^ In this work, we found that MALAT1 enhances the stability of ACSF2 mRNA via direct interaction with RNA‐binding protein FUS. Our results demonstrate that MALAT1 maintains the stability of ACSF2 mRNA via interaction with FUS, which triggered ferroptosis in septic AKI.

However, this study has some limitations. First, due to the poor transfection efficiency of shACSF2, we could not determine the role of ACSF2 silencing in ferroptosis in HK‐2 cells. Second, according to a previous study, FUS protein could be ubiquitinated and destabilized by lncRNA SOX2OT.^30^ Whether lncRNA MALTA1 can reduce FUS expression via promoting its ubiquitination and degradation needs further validation. Third, although we demonstrated that FUS binds to and stabilizes the mRNA of the ferroptosis‐related gene ACSF2, the exact regulatory mechanism of FUS in its target ACSF2 mRNA remains unclear. Fourth, MALTA1 silencing could reduce FUS mRNA level, but not affected FUS mRNA stability, MALTA1 might regulate FUS expression via other mechanisms. For example, acting as a ceRNA, lncRNA can regulate its target gene expression via sponging miRNA.^31^ Whether MALTA1 can regulate FUS mRNA expression via sponging miRNAs needs to be explored in the future. Finally, while FUS was mainly expressed in the mitochondria of HK‐2 cells and MALAT1 deficiency repressed both mitochondrial and cytoplasmic FUS expression, the upregulation of ACSF2 in the cytoplasm might be modulated by other complicated mechanisms that require further investigation.

Taken together, our results demonstrated that the abnormal high expression of MALAT1 promotes the inflammatory response and ferroptosis during septic AKI progression. Mechanistically, MALAT1 induces ferroptosis by interacting with FUS to enhance the stability of ACSF2 mRNA. These findings uncover a novel mechanism through which MALAT1 increases ACSF2 mRNA stability, thereby accelerating septic AKI development. Therefore, MALAT1 might be a therapeutic target for overcoming septic AKI.

## CONFLICT OF INTEREST STATEMENT

The authors declare no conflict of interest.

## Supporting information


**FIGURE S1:** ACSF2 knockdown repressed ferroptosis in LPS‐stimulated HK‐2 cells. (A) RT‐qPCR analysis of ACSF2 expression in HK‐2 cells transfected with shNC or shACSF2. LPS‐exposed HK‐2 cells were transfected with shNC or shACSF2. (B) HK‐2 cell viability was determined by CCK‐8 assay. (C‐D) MDA and GSH levels in HK‐2 cells were measured. (E) Intracellular ROS level in HK‐2 cells was assessed by flow cytometry. (F) Fe^2+^ level in HK‐2 cells was measured. *p < 0.05, **p < 0.01, ***p < 0.001. *n* = 3.
**FIGURE S2:** Effect of MALAT1 on FUS and ACSF2 mRNA stability. (A) HK‐2 cells were infected with LV‐shNC or LV‐shMALAT1, and FUS mRNA stability was evaluated by RT‐qPCR. (B) HK‐2 cells were transfected with vector or MALAT1 overexpression plasmid combined with siNC or siFUS, and ACSF2 expression and stability were assessed by RT‐qPCR. *p < 0.05, **p < 0.01, ***p < 0.001. *n* = 3.

## Data Availability

All data generated or analyzed are included in this article. Further inquiries can be directed to the corresponding author.
