# Correction to “Long non‐coding RNA MALAT1 triggers ferroptosis via interaction with FUS to enhance ACSF2 mRNA stabilization in septic acute kidney injury”

**DOI:** 10.1002/kjm2.12947

**Published:** 2025-02-13

Duan ZB, Zheng JF, Huang SY, Hu LL. Long non‐coding RNA MALAT1 triggers ferroptosis via interaction with FUS to enhance ACSF2 mRNA stabilization in septic acute kidney injury. Kaohsiung J Med Sci. 2024;40(11):972–84. https://doi.org/10.1002/kjm2.12898

Due to clerical error, in Figure 4, the data presented in 4F and 4G were inadvertently duplicated. The corrected figure is provided below.

**Figure:**
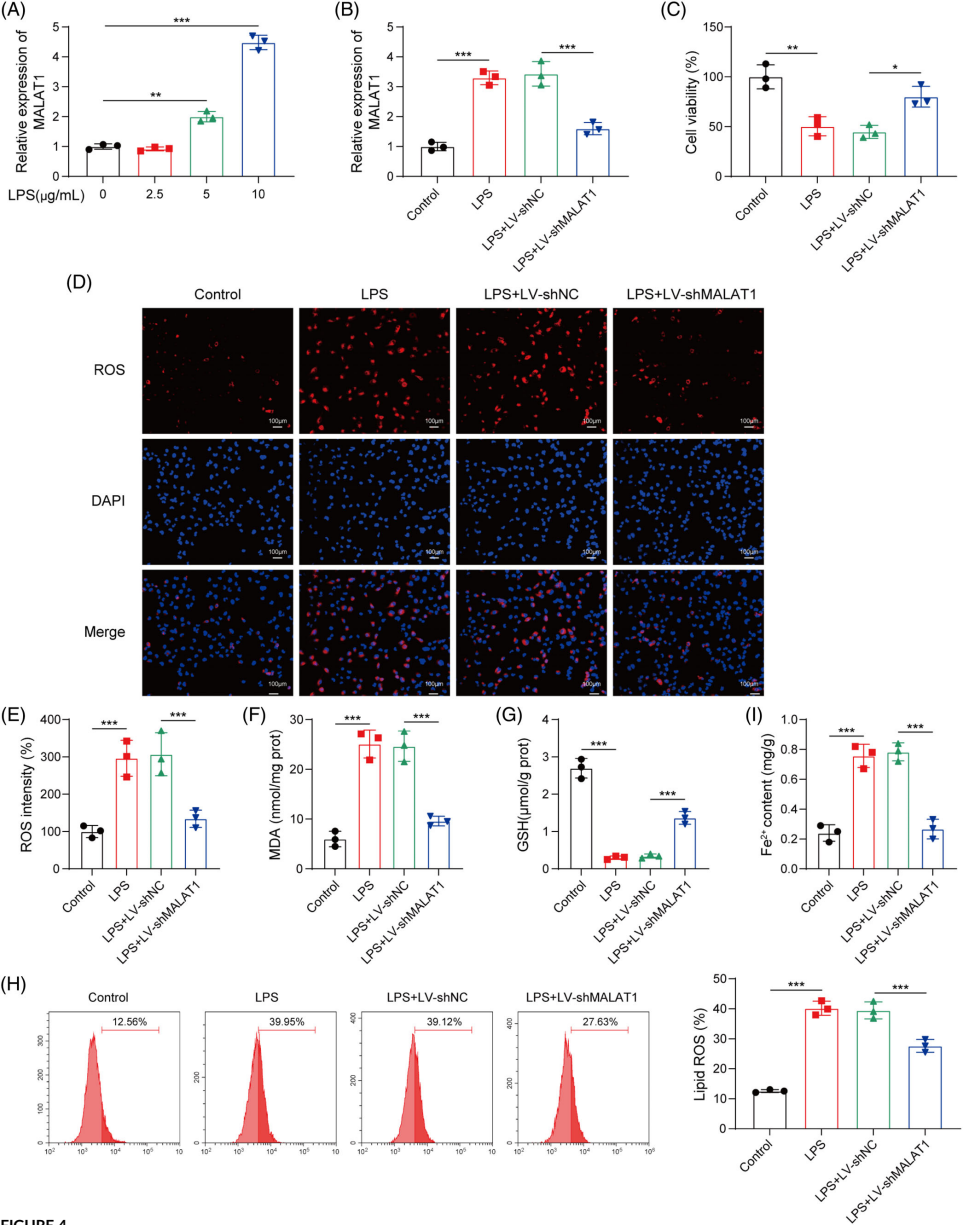
FIGURE 4

We apologize for this error. The authors confirmed that all results and conclusions of this article remain unchanged.

