# Supplementary material for: Long non‐coding RNA MALAT1 triggers ferroptosis via interaction with FUS to enhance ACSF2 mRNA stabilization in septic acute kidney injury
Source: Kaohsiung J Med Sci. 2024 Oct 10;40(11):972–84. doi: 10.1002/kjm2.12898 (PMC11895079; doi:10.1002/kjm2.12898)
Supplement: Supplementary file 1 — FIGURE S1: ACSF2 knockdown repressed ferroptosis in LPS‐stimulated HK‐2 cells. (A) RT‐qPCR analysis of ACSF2 expression in HK‐2 cells transfected with shNC or shACSF2. LPS‐exposed HK‐2 cells were transfected with shNC or shACSF2. (B) HK‐2 cell viability was determined by CCK‐8 assay. (C‐D) MDA and GSH levels in HK‐2 cells were measured. (E) Intracellular ROS level in HK‐2 cells was assessed by flow cytometry. (F) Fe2+ level in HK‐2 cells was measured. *p < 0.05, **p < 0.01, ***p < 0.001. n = 3. FIGURE S2: Effect of MALAT1 on FUS and ACSF2 mRNA stability. (A) HK‐2 cells were infected with LV‐shNC or LV‐shMALAT1, and FUS mRNA stability was evaluated by RT‐qPCR. (B) HK‐2 cells were transfected with vector or MALAT1 overexpression plasmid combined with siNC or siFUS, and ACSF2 expression and stability were assessed by RT‐qPCR. *p < 0.05, **p < 0.01, ***p < 0.001. n = 3. [file KJM2-40-972-s001.docx]

**Supplementary figure legends**

**
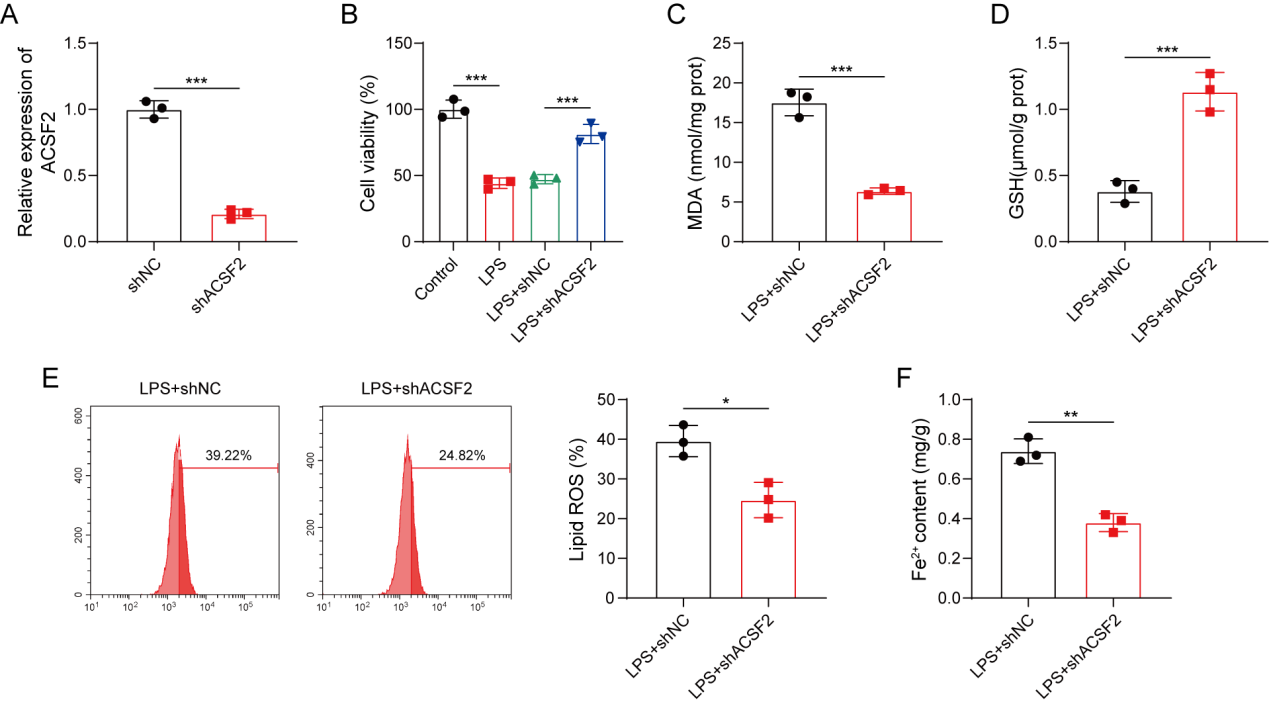
**

**Figure S1. ACSF2 knockdown repressed ferroptosis in LPS-stimulated HK-2 cells.** (A) RT-qPCR analysis of ACSF2 expression in HK-2 cells transfected with shNC or shACSF2. LPS-exposed HK-2 cells were transfected with shNC or shACSF2. (B) HK-2 cell viability was determined by CCK-8 assay. (C-D) MDA and GSH levels in HK-2 cells were measured. (E) Intracellular ROS level in HK-2 cells was assessed by flow cytometry. (F) Fe^2+^ level in HK-2 cells was measured. *P < 0.05, **P < 0.01, ***P < 0.001. n=3.

**
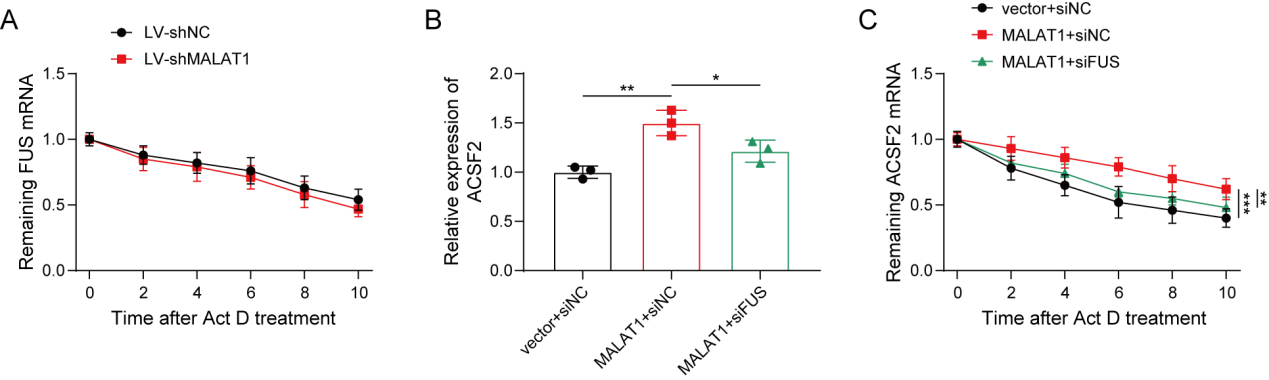
**

**Figure S2.** **Effect of** **MALAT1 on FUS and** **ACSF2 mRNA stability.** (A) HK-2 cells were infected with LV-shNC or LV-shMALAT1, and FUS mRNA stability was evaluated by RT-qPCR. (B) HK-2 cells were transfected with vector or MALAT1 overexpression plasmid combined with siNC or siFUS, and ACSF2 expression and stability were assessed by RT-qPCR. *P < 0.05, **P < 0.01, ***P < 0.001. n=3.
